# Macroscopic and Microscopic Investigation of Gypsum Slag Cement-Stabilized Recycled Aggregate Base Layers

**DOI:** 10.3390/ma17061450

**Published:** 2024-03-21

**Authors:** Changdong Zhou, Pengcheng Shi, Hao Huang, Junan Shen

**Affiliations:** 1 Jiangsu Technology Industrialization and Research Center of Ecological Road Engineering, Suzhou University of Science and Technology, Suzhou 215011, China; 2 Department of Civil Engineering and Construction, Georgia Southern University, Statesboro, GA 30458, USA

**Keywords:** gypsum slag cement, recycled aggregate, compressive strength, tensile strength

## Abstract

The purpose of this study is to investigate the macro and micro properties of stabilized recycled aggregate base layers using gypsum slag cement (GSC) and compare them with ordinary Portland cement (OPC). To achieve this, four levels of recycled aggregate content (0%, 50%, 60%, 70%) and three levels of binder materials (3.5%, 4.5%, 5.5%) were designed, where the binding materials included OPC and GSC. When GSC is used as the binding material with 0% recycled content, two scenarios for the ratio of slag to activator are considered: 4:1 and 4:2. For recycled content of 50%, 60%, and 70%, only the 4:1 ratio is considered. The macro-mechanical properties of the composite material were studied through compaction tests, unconfined compressive strength tests, and indirect tensile strength tests. Microscopic properties were investigated through X-ray diffraction (XRD) and scanning electron microscopy (SEM). Macroscopic test results indicate that, at an equal binder content, GSC exhibits a higher moisture content and maximum dry density compared to OPC. Moreover, the unconfined compressive strength and indirect tensile strength of GSC are higher than those of OPC. Microscopic test results reveal that the hydration products of both binding materials are essentially similar; however, under identical curing conditions, the hydration products of GSC are more abundant than those of OPC.

## 1. Introduction

Highway engineering relies heavily on the use of substantial amounts of sand and gravel. However, the increasing global emphasis on environmental conservation has led to a scarcity of natural materials, causing a surge in prices. The recycling of waste concrete aggregates presents a viable alternative to natural sand and gravel, offering a cost reduction and minimizing environmental impact. Therefore, investigating the performance of recycled concrete aggregates in road applications not only addresses the issue of supply shortages but also contributes significantly to environmental protection. Additionally, the utilization of slag, a byproduct of iron smelting in blast furnaces, is crucial for both environmental protection and waste management.

Research on recycled aggregates has a longstanding history globally, yielding numerous research findings over time. In general, recycled aggregates demonstrate a performance inferior to that of natural aggregates. Consequently, scholars worldwide have extensively investigated methods to enhance the various performance aspects of recycled aggregates.

Some scholars have experimented with immersing recycled aggregates in a 10% silica fume solution, resulting in an improved compressive strength of the concrete formulated with these treated aggregates after 28 days [1]. Further refinement of this method has been achieved through the implementation of an accelerated carbonation treatment on aggregates at a 50% carbon dioxide concentration level [2]. Additionally, nano SiO_2_ has been employed by some scholars to enhance the overall performance of recycled aggregate concrete [3].

As research on recycled aggregates continues to expand, various treatment methods have been identified to improve the microstructure of recycled aggregates. These methods include chemical or carbon treatments, combined treatments, thermal chemical techniques to remove cement paste from fine recycled aggregates, and the application of recycled materials in soil improvement [4,5,6,7]. In an effort to promote economic and environmental sustainability in the construction industry and to optimize the utilization of recycled concrete aggregates, scholars have proposed a novel model. This model aims to predict the compressive strength and modulus of elasticity of concrete prepared using treated recycled concrete aggregates and can be applied in the design of concrete structures incorporating treated recycled aggregates [8].

Apart from the investigation into recycled aggregates, numerous scholars have conducted in-depth explorations into cementitious materials, with a particular emphasis on diverse activation methods for slag. Research findings indicate that granulated blast furnace slag exhibits sluggish hydration rates when in contact with pure water, primarily due to the rapid formation of an impermeable layer of aluminosilicate film on the particle surface, impeding further hydration. However, alkali activators have been identified to accelerate the dissolution of slag glass, facilitating the hydration process. Consequently, scholars have extensively studied various alkali activators, including NaOH, Na_2_CO_3_, Na_2_O·nSiO_2_, and Na_2_SO_4_ [9,10,11,12,13]. Furthermore, methodologies involving the activation of granulated blast furnace slag using sulfate have been explored. Sulfate activators, such as gypsum, react with Al_2_O_3_ to yield ettringite, thereby enhancing early strength. The blend consisting of 10–20% calcium sulfate (primarily in anhydrite form), 75–90% slag, and a small portion of ordinary silicate cement is termed sulfoaluminate cement (also recognized as gypsum slag cement). Over time, research on sulfoaluminate cement has progressed extensively, encompassing the activation of various slag types, the incorporation of phosphogypsum into sulfoaluminate cement, and the utilization of volcanic ash in sulfoaluminate cement preparation [14,15,16,17,18,19,20,21,22,23].

In conclusion, the utilization of recycled concrete aggregates and slag in highway construction offers a sustainable solution to the challenges posed by the scarcity of natural materials and environmental concerns. Research efforts globally have focused on enhancing the properties of recycled aggregates and optimizing the activation of slag for improved performance in cementitious materials. These developments contribute significantly to the sustainability and environmental compatibility of road construction practices. Further research and advancements in technology are essential to unlock the full potential of these alternative materials in the field of highway engineering.

This study presents a comparative analysis of the mechanical properties and microstructural features of stabilized recycled concrete aggregates using a novel sulfoaluminate cement and OPC.

## 2. Materials and Methods

### 2.1. Materials

#### 2.1.1. OPC

Cement assumes a pivotal role as an essential binding material in semi-rigid base layers, crucial for satisfying diverse performance criteria. In this investigation, Tian Shan PO 42.5 cement, meticulously formulated for road applications, is selected for examination. The various performance indicators of this cement undergo rigorous testing in adherence to the standardized specifications outlined in the “Specification for Portland Cement” (ASTM C150/C150M-22) [24] and the “Standard Test Methods for Chemical Analysis of Hydraulic Cement” (ASTM C114-22) [25]. Detailed experimental outcomes are elucidated in Table 1 and Table 2. 

#### 2.1.2. GSC

The gypsum slag cement utilized in this experiment comprises two primary components: granulated blast furnace slag and an activator agent.

The slag employed in this experimental study is granulated blast furnace slag obtained from Beijing Zhihua Technology Co., Ltd., Beijing, China. Its predominant chemical composition is delineated in Table 3, with a specific surface area measuring 525 m^2^/kg. It is noteworthy that the specific surface area of the slag plays a significant role in influencing the hydration reaction, whereby a larger specific surface area indicates a greater extent of surface involvement in the reaction. The activator comprises primarily sulfoaluminate cement clinker and gypsum, both sourced from Beijing Zhihua Technology Co., Ltd.

#### 2.1.3. Aggregates

The recycled aggregates (RA) utilized in this study were procured from the Suzhou Construction Waste Recycling Limited Company, Suzhou, China, while the natural aggregates (NA), comprising limestone, were obtained from Suzhou Taifa Building Materials Co., Ltd., Suzhou, China. A total of six material gradations were employed, encompassing recycled coarse aggregate (15–31.5 mm), recycled intermediate aggregate (5–15 mm), recycled fine aggregate (0–5 mm), natural coarse aggregate (15–25 mm), natural intermediate aggregate (5–15 mm), and natural fine aggregate (0–5 mm). Adhering to the specifications outlined in the JTG E42-2005 “Test Code for Aggregate for Highway Engineering” [26], sieve tests and pertinent physical–mechanical performance assessments were conducted on both natural and recycled aggregates. The detailed test outcomes are presented in Table 4 and Table 5.

### 2.2. Methods

#### 2.2.1. Mix Proportion Design

The designed combinations for the mixed materials in this experiment encompassed two aspects: the recycled material content was set at 0%, 50%, 60%, and 70%, and each recycled content underwent testing with three different cementitious materials contents—3.5%, 4.5%, and 5.5%. When the recycled material content is set at 0%, 50%, 60%, and 70%, each level of recycled content undergoes testing with an activator to slag ratio of 1:4. Additionally, when the recycled material content is 0%, further experiments are conducted with an 2:4 activator–slag. Throughout the actual experimental proceedings, it was observed that when the recycled material content was 0% and the activator–slag ratio was 1:4, the 7-day unconfined compressive strength of the mixture nearly approached zero. In response, the activator ratio was increased to 2:4, leading to subsequent testing that demonstrated a notable increase in strength. Consequently, in the subsequent experiments where the recycled material content was 0%, the activator–slag ratio was consistently maintained at 2:4.

#### 2.2.2. Gradation Selection

In adherence to the Technical Specifications for Highway Pavement Base Construction (JTG/TF20-2015) [27], the gradation range for the mixture is determined based on the recommended C-B-3 gradation. The detailed gradation data for this experiment are provided in Table 6.

#### 2.2.3. Compaction Test

Accordance with the testing procedures outlined in the JTG E51-2009 “Test Code for Stabilized Materials in Highway Engineering with Inorganic Binders” based on the T0804-1994 [28] standard, compaction tests were conducted. T optimal moisture content and maximum dry density were determined for each type of mixture.

The experimental method is as follows: For each cement dosage, at least five specimens of approximately 5.5 kg each are obtained using the quartering method. Subsequently, five different moisture contents are selected around the estimated optimum moisture content, varying by approximately 0.5% to 1.5% increments. Prior to experimentation, water is added to the specimens and thoroughly mixed. The mixture is then placed in plastic bags for soaking, with a minimum soaking time of 2 h. Before conducting the compaction test, cement is added to the mixture and thoroughly mixed. The compaction test must be completed within 1 h. Subsequently, the compaction test is carried out according to the parameters specified in Table 7. The moisture content–dry density curve is plotted based on the compaction data of each group, and the maximum dry density and optimum moisture content of each group are calculated based on the fitted equation.

#### 2.2.4. Unconfined Compressive Strength

Following the procedures outlined in the JTG E51-2009 “Test Methods for Stabilized Materials in Highway Engineering with Inorganic Binders,” particularly T0843-2009 and T0805-1994 [28], specimens were meticulously crafted and subjected to a curing regime, subsequently undergoing unconfined compressive strength testing.

In this study, cylindrical molds with dimensions of a diameter × height = 150 mm × 150 mm were employed. Subsequently, a standardized specimen mass was calculated based on the specimen volume, maximum dry density, and optimal moisture content for each group. Considering a compaction degree of 98%, the weights of the aggregates, water, and cement were then determined using formulas provided in the specifications. Prior to molding the cylindrical specimens, the water and aggregates were uniformly mixed and allowed to soak for a minimum of 2 h. Following this, cement was added, mixed thoroughly, and the mixture was molded under pressure. 

#### 2.2.5. Indirect Tensile Strength Test

Following the methodologies outlined in JTG E51-2009, which provides technical specifications for testing inorganic stabilized materials in highway engineering, specifically referring to T0843-2009 and T0806-1994 [28], specimens were meticulously prepared. These specimens underwent a curing process for both 7 and 28 days, after which they were subjected to an indirect tensile strength test.

#### 2.2.6. XRD Test

The X-ray diffractometer utilized in this investigation is the Bruker D8 Advance, crafted by Bruker in Bremen, Germany. The instrument was configured with a scanning range spanning from 10° to 90° (2θ), employing a step size of 0.2024962, and comprising a total of 3904 steps. Notably, a Cu target was selected as the X-ray source for the experimental procedures.

## 3. Experimental Results and Analysis

### 3.1. Compaction Test

Table 8 illustrates the results of the compaction tests, revealing trends in the optimal moisture content: With a constant amount of OPC, the optimum moisture content increases with the rise in recycled aggregate content. For instance, when the OPC content is 3.5%, and the RA content varies from 0% to 70%, the optimum moisture content increases from 4.6% to 7.8%. Keeping the recycled aggregate content constant, an increase in the OPC content results in a higher optimum moisture content. For instance, with a 0% RA content, as the OPC content increases from 3.5% to 5.5%, the optimum moisture content rises from 4.6% to 5.2%. Under the same RA content and cementitious material content, the GSC exhibits a higher optimum moisture content compared to the OPC, with an increase of approximately 0.4%. With a constant GSC content, the optimum moisture content increases with higher RA content. Similarly, with a constant RA content, an increase in GSC content leads to a rise in optimum moisture content, mirroring the trends observed with OPC.

Table 9 provides the maximum dry density obtained from the compaction tests. Key findings include: With a constant OPC content, the maximum dry density demonstrates a diminishing trend with an escalating RA content. While keeping recycled RA content unaltered, amplifying the OPC content results in an augmented maximum dry density. In scenarios where the RA and cementitious material contents are identical, GSC asserts its dominance, exhibiting a higher maximum dry density by approximately 0.1 g/cm^3^ in contrast to OPC. At a stable GSC content, the maximum dry density experiences a decline in the presence of heightened RA content. Similarly, maintaining RA content at a constant and augmenting GSC content induces a rise in the maximum dry density, mirroring the patterns observed in OPC situations.

The analysis of the experimental results above indicates that, when the dosage of recycled aggregates and cementitious materials remains constant, both the maximum dry density and optimum moisture content of gypsum slag cement exceed those of ordinary Portland cement under similar conditions. This phenomenon can be attributed to the larger specific surface area of gypsum slag cement compared to ordinary Portland cement. The specific surface area of the gypsum slag cement used in this experiment is 525 m^2^/kg, whereas that of the ordinary Portland cement is 339 m^2^/kg. During the hydration process, cement particles with larger specific surface areas possess more surface area, thus requiring more moisture to wet and envelop each particle to ensure the fluidity and workability of the concrete. Consequently, the optimum moisture content of gypsum slag cement surpasses that of ordinary Portland cement. Consequently, during compaction tests, an increase in moisture content under identical volume conditions leads to enhanced concrete quality and, consequently, an increase in density.

### 3.2. Seven-Day Unconfined Compressive Strength

The detailed weights and compositions of the specimens in each group are delineated in the following Table 10, as per academic convention.

**Figure 1:**
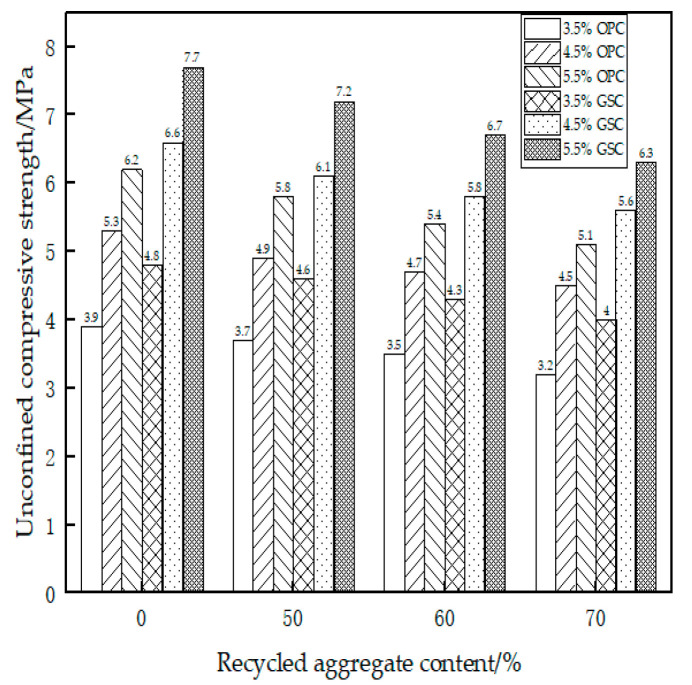
Seven-day unconfined compressive strength test.

Table 11 delineates the unconfined compressive strength acquired from specimens meticulously crafted and subjected to a seven-day curing period, grounded in the optimal moisture content and maximum dry density ascertained through the compaction test. Figure 1 elucidates a column chart extrapolated from the data in Table 11. An analysis of Table 11 and Figure 1 reveals the following observations: Within the same recycled material and binder composition, the unconfined compressive strength of GSC surpasses that of OPC. For instance, with a recycled material content of 60% and a binder content of 4.5%, the unconfined compressive strength is 4.7 MPa for OPC and 5.8 MPa for gypsum slag cement, signifying an increase of approximately 25%. When the GSC content remains constant, an augmentation in the recycled aggregate content leads to a diminution in the seven-day unconfined compressive strength. For instance, with the GSC content at 3.5% and the recycled material content at 0%, 50%, 60%, and 70%, the unconfined compressive strength descends from 4.8 MPa to 4 MPa, indicating a reduction of 16%. Holding a consistent recycled material content, an escalation in GSC corresponds to an augmentation in the unconfined compressive strength. For example, with the recycled material content at 0%, the GSC content at 3.5%, 4.5%, and 5.5%, the unconfined compressive strength ascends from 4.8 MPa to 7.7 MPa, representing an increase of approximately 60%. Analogous to GSC, the unconfined compressive strength of OPC diminishes with an upswing in recycled material content when the cement content is sustained. Conversely, with a constant RA content, an elevation in cement content leads to a surge in unconfined compressive strength.

Through the compaction test and the seven-day unconfined compressive strength test, it is discernible that, at a cement content of 4.5%, OPC aligns with the requisites for foundational road construction expounded in the Technical Specifications for Highway Base Construction (JTG/T F20-2015) [27] for high-speed highways and first-class roads enduring heavy traffic (4–6 MPa). GSC, at a content of 3.5%, already meets these road-grade specifications. Furthermore, with a GSC content of 4.5%, it satisfies the criteria for foundational road construction in conditions of extremely heavy and special heavy traffic on high-speed highways and first-class roads (5–7 MPa).

### 3.3. 28-Day Unconfined Compressive Strength

**Figure 2:**
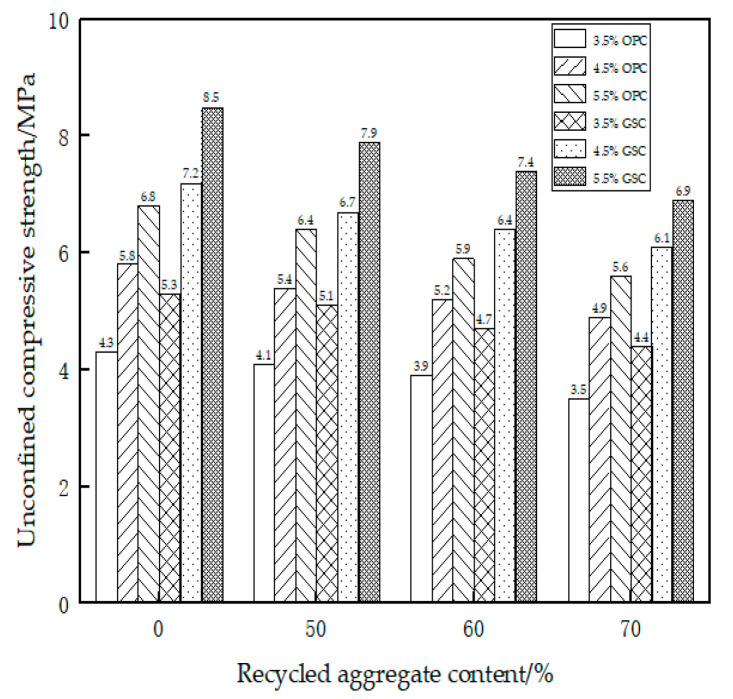
The 28-day unconfined compressive strength test.

The specimens were prepared and tested using the same methods as described above for the 28-day unconfined compressive strength. The experimental results are presented in Table 12 and Figure 2.

Table 12 depicts the 28-day unconfined compressive strength under varying levels of recycled material content. Observations from the table reveal the following trends: (1) Whether employing OPC or GSC, the 28-day unconfined compressive strength exhibits a pattern consistent with the earlier-discussed 7-day results. (2) Post 28 days of curing, the unconfined compressive strength of GSC consistently surpasses that of OPC, showcasing an approximate 25% superiority. (3) Following the extended 28-day curing period, both OPC and GSC manifest strengthened performance, with an approximately 10% enhancement in unconfined compressive strength.

### 3.4. Indirect Tensile Strength Test

The comprehensive outcomes of this testing are meticulously detailed in Table 13 and Table 14.

**Figure 3:**
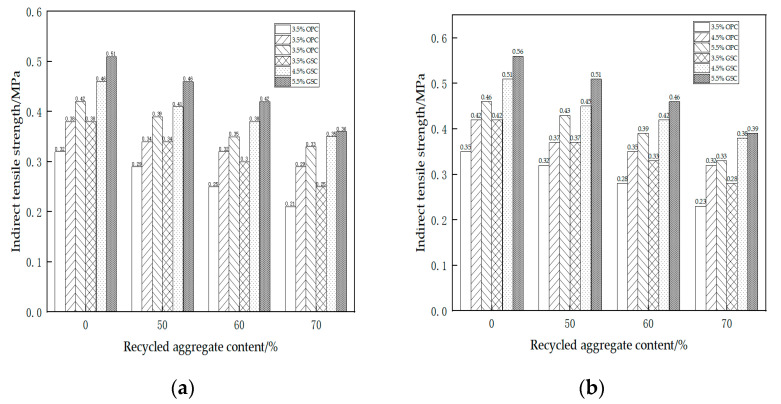
Indirect tensile strength. (**a**) After 7 days; (**b**) After 28 days.

Table 13 presents the results of the seven-day indirect tensile strength tests, and Figure 3a provides a visual representation of the data. The findings reveal several key observations: Under consistent proportions of recycled materials and binder content, GSC demonstrates a superior indirect tensile strength compared to OPC. For instance, with a recycled material content of 60% and a binder content of 4.5%, the indirect tensile strength of OPC is 0.32 MPa, while that of GSC is 0.38 MPa, indicating a notable enhancement of approximately 19%. With a constant GSC content, the seven-day indirect tensile strength decreases as the RA content increases. For example, with a GSC content of 3.5% and a recycled material content varying from 0% to 70%, the indirect tensile strength decreases from 0.38 MPa to 0.25 MPa, marking a substantial reduction of 34%. Maintaining a consistent recycled material content, an increase in GSC content leads to a corresponding rise in the seven-day indirect tensile strength. For instance, with a recycled material content of 0%, a GSC content of 3.5%, 4.5%, and 5.5%, the indirect tensile strength increases from 0.38 MPa to 0.51 MPa, representing an approximately 34% improvement. The behavior of OPC aligns with that of GSC. When the content of OPC is constant, an increase in recycled material content results in a decrease in the indirect tensile strength. Similarly, with a constant RA content, an increase in the cement content leads to an enhancement in the indirect tensile strength.

Moving to Table 14, which showcases the results of the 28-day indirect tensile strength tests, and Figure 3b, depicting the corresponding graphical representation, we draw the following conclusions: The patterns observed in both OPC and GSC for the 28-day tests mirror those identified in the 7-day tests. After 28 days of curing, the indirect tensile strength of GSC remains notably higher than that of OPC, displaying an increase of approximately 20%. The strength of both OPC and GSC experiences a positive growth, with an approximate 10% increase in indirect tensile strength after 28 days of curing.

### 3.5. XRD Test Results and Analysis

**Figure 4:**
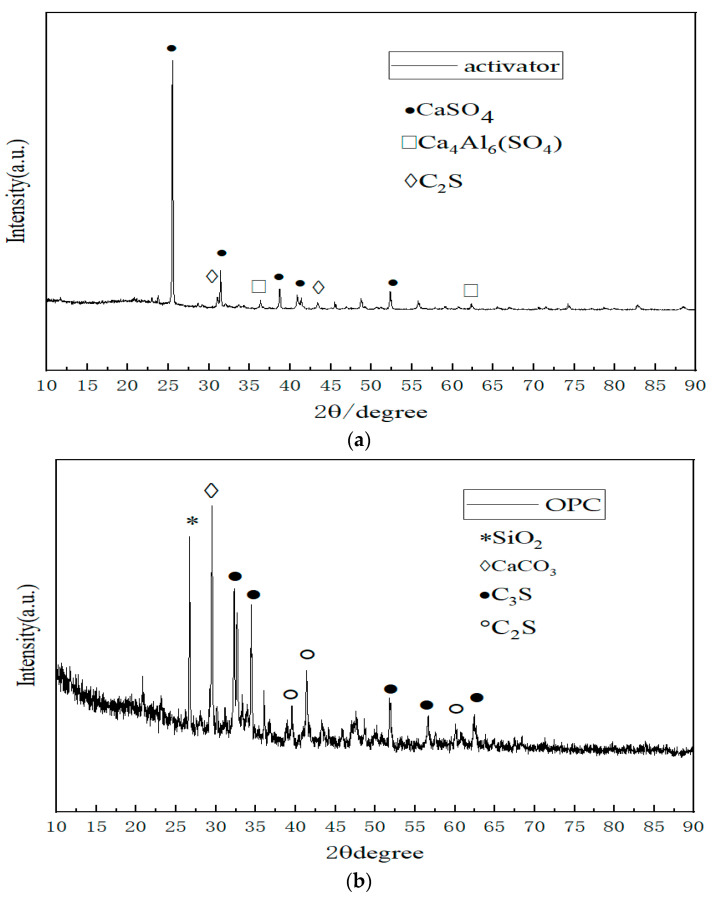
XRD Pattern. (**a**) Activator; (**b**) OPC.

Figure 4 illustrate the XRD patterns of the activator and cement, respectively. An analysis of these spectra reveals that the primary constituents of the activator are gypsum (CaSO_4_) and sulfoaluminate cement clinker, characterized by predominant phases of C_2_S and Ca_4_Al_6_(SO_4_). In contrast, the ordinary Portland cement is primarily composed of SiO_2_, CaCO_3_, C_3_S, and C_2_S, as evident from the XRD results.

**Figure 5:**
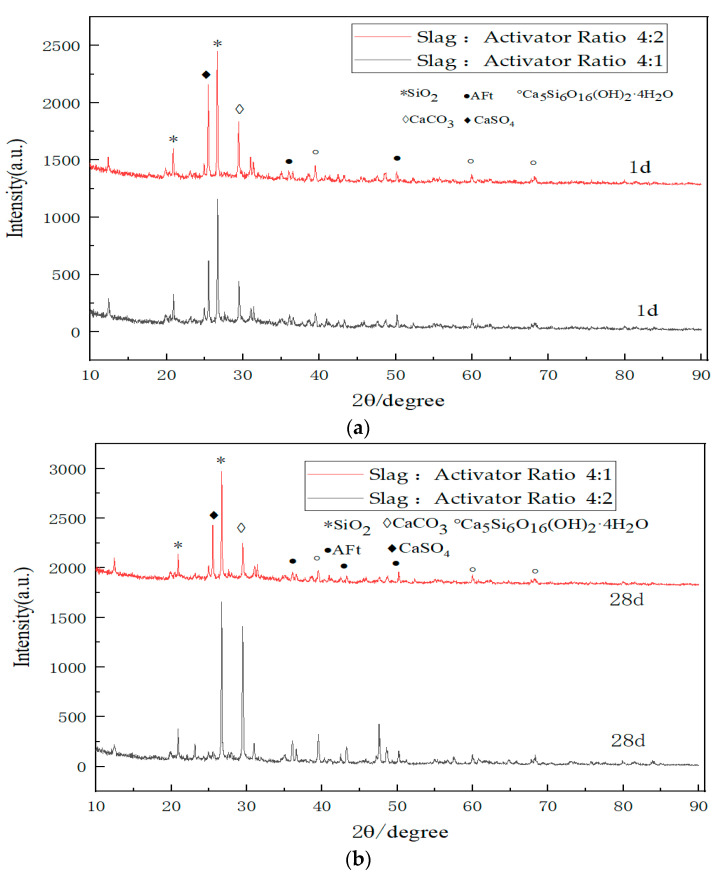
XRD pattern. (**a**) Hydration reaction time of GSC with a 0% RA content for one day; (**b**) Hydration reaction time of GSC with a 0% RA content for 28 days.

Figure 5a depicts the XRD patterns of GSC hydration after one day, utilizing exclusively new aggregates. The following conclusions can be drawn from the analysis: The hydration products are predominantly consistent after one day when the slag–activator ratio is either 4:1 or 4:2. The heightened proportion of the activator results in a more pronounced presence of CaSO_4_ characteristic peaks in the XRD pattern when the slag–activator ratio is 4:2, as compared to the ratio of 4:1. Both ratios exhibit the presence of C-S-H gel and AFt in the XRD patterns. However, a nuanced observation suggests that, when the slag–activator ratio is 4:2, the intensity of the peaks corresponding to the C-S-H gel and AFt appears to be marginally greater than in the case of a 4:1 ratio.

Figure 5b presents the X-ray diffraction (XRD) pattern of GSC after 28 days of hydration with entirely new aggregates. From the graph, the following observations can be made: The products are essentially consistent when the slag–activator ratio is 4:1 or 4:2 after 28 days of hydration. After 28 days of hydration, the characteristic peaks of various hydration products when the slag–activator ratio is 4:2 are significantly stronger than those when the ratio is 4:1.

Combining the insights from Figure 5a,b, it can be concluded that in the early stages of GSC hydration, the slag–activator ratio has a relatively minor impact on the hydration reaction. However, with the passage of time, the hydration reaction is notably sluggish when the slag–activator ratio is 4:1, leading to a virtually zero unconfined compressive strength at the macroscopic level. This phenomenon is attributed to the hydration mechanism of GSC, wherein gypsum serves as the sulfate activator for slag, and the sulfoaluminate cement clinker acts as the alkaline activator. Under the influence of these two activators, granulated blast furnace slag is stimulated to hydrate. The key to this activation lies in achieving a certain level of alkalinity in the entire system during the reaction process. If the alkalinity of the entire system is insufficient, i.e., in the presence of fewer OH- ions, the surface structure of the slag cannot be disrupted. This prevents the precipitation of active SiO_2_ and active Al_2_O_3_, leading to a limited formation of Al^3+^ and Si^4+^ ions. Consequently, the subsequent formation of C-S-H gel and ettringite becomes compromised. Therefore, by increasing the proportion of the activator, the OH- ions provided by the hydration of cement clinker increase, reaching the alkalinity required for GSC hydration. The hydration reaction of GSC continues normally under the condition of entirely new aggregates, resulting in a macroscopic unconfined compressive strength reaching normal values and even surpassing the strength of specimens made with OPC.

**Figure 6:**
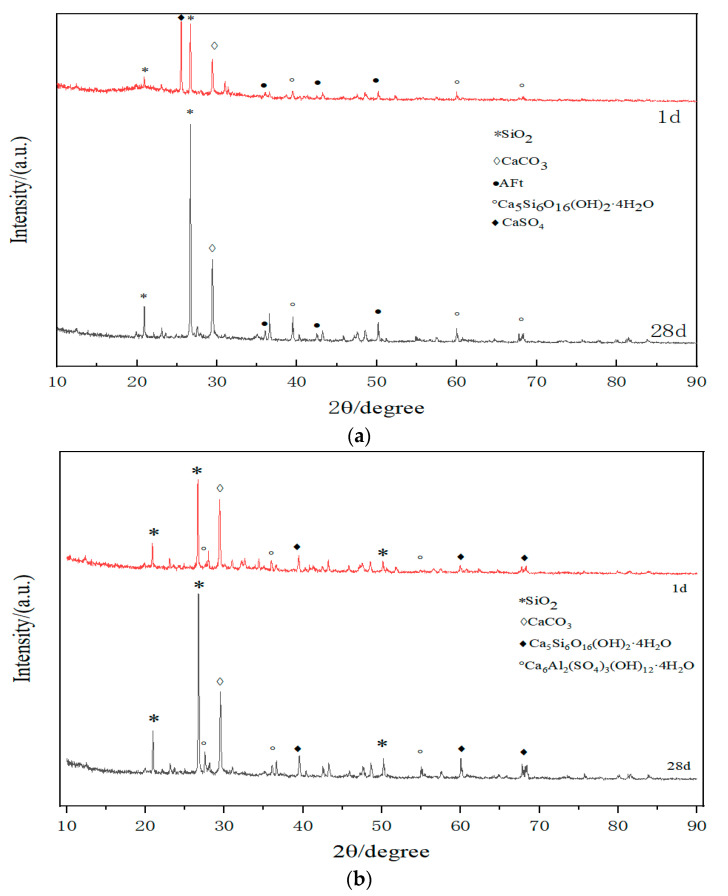
XRD Pattern. (**a**) Hydration reaction times of GSC with a 70% RA content at 1 day and after 28 days; (**b**) Hydration reaction times of OPC with a 70% RA content at 1 day and after 28 days.

Figure 6a illustrates the XRD analysis of hydration products at different ages for a 4.5% GSC with a 70% recycled material content. The hydration mechanism of GSC involves the initial dissolution of gypsum, a sulfoaluminate cement clinker, and other components within the system, resulting in the release of OH^−^, Ca^2+^, SO_4_^2−^, and a modest quantity of Si^4+^ and Al^3+^ ions. This initiates the formation of limited amounts of hydrated calcium silicate gel and hydrated calcium aluminate gel.

Following this, the hydrated calcium aluminate gel reacts with the SO_4_^2−^ ions in the system, giving rise to ettringite. However, due to the constrained availability of the cement clinker, there is a deficiency of Si^4+^ and Al^3+^ ions in the system, impeding further hydration reactions. Nevertheless, the preceding hydration reactions elevate the alkalinity of the entire system. Under the influence of OH^−^ ions, the Si-O and Al-O in the slag undergo depolymerization-repolymerization reactions, hastening the dissolution of Si^4+^ and Al^3+^ ions. This leads to the liberation of Ca^2+^ ions into a free state, participating in reactions within the alkaline environment to form a C-S-H gel and hydrated calcium aluminate gel.

Concurrently, the presence of calcium sulfate augments the sulfate content in the binder material, engaging in reactions with hydrated calcium aluminate to form ettringite. As per the elucidated hydration mechanism, Figure 6a reveals distinct features. At one day of hydration, the pronounced characteristic peak of CaSO_4_ suggests the incomplete participation of gypsum in the hydration reaction. Although the characteristic peaks of hydrated calcium silicate gel and AFt are discernible, their intensities are comparatively subdued. With the progression of hydration, particularly after 28 days of curing, a notable reduction in the intensity of the CaSO_4_ peak is observed in contrast to the 1-day hydration period. Conversely, the characteristic peaks of hydrated calcium silicate gel and ettringite exhibit a significant augmentation in intensity. This phenomenon is macroscopically manifested as an elevation in compressive strength with the extension of the curing period.

The outcomes reveal a noteworthy observation: under a 4:1 ratio of slag-to-activator, GSC fails to achieve the requisite strength in the absence of recycled materials (0% content). However, a modification in the 4:2 slag–activator rectifies this deficiency, enabling the attainment of the desirable strength. Intriguingly, in scenarios where the recycled material content is 50%, 60%, and 70%, GSC demonstrates strength even under the 4:1 ratio.

This phenomenon can be elucidated by the inclusion of recycled fine powder in the recycled aggregates, which contains substances such as CaOH capable of supplying additional OH- ions. This augmentation in OH- ions enhances the overall alkalinity of the reaction system, facilitating sustained reactions within gypsum slag cement even when subjected to a 4:1 ratio.

Figure 6b depicts the XRD analysis of the hydration products at various stages for 4.5% OPC with a 70% recycled material content. The hydration mechanism of OPC involves the predominant early stage hydration of C_3_S, resulting in the formation of hydrated calcium silicate (C-S-H) and crystalline calcium hydroxide. The hydration of C_2_S progresses at a slower rate, yielding primarily C-S-H and a minor amount of calcium hydroxide. C_3_A exhibits the fastest reaction rate, with the primary products being ettringite (AFt) and monosulfate (AFm).

In accordance with the aforementioned reaction mechanism, characteristic peaks of C-S-H and ettringite are observable after one day of hydration, albeit with a reduced intensity. However, after 28 days of hydration, the characteristic peaks of C-S-H and ettringite exhibit an increased intensity compared to the 1-day hydration period. This is reflected at a macroscopic level by an elevation in the unconfined compressive strength.

Analyzing the XRD patterns of GSC and OPC, it can be inferred that the hydration products of both predominantly consist of ettringite and C-S-H gel, with no significant differences between the two in terms of their hydration products.

### 3.6. SEM Results and Analysis

**Figure 7:**
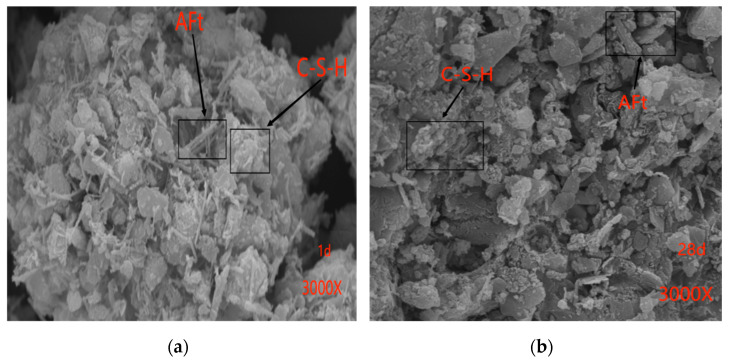
Scanning electron microscopy images. (**a**) Hydration reaction time of OPC with a 70% RA content for one day; (**b**) Hydration reaction time of OPC with a 70% RA content for 28 days.

**Figure 8:**
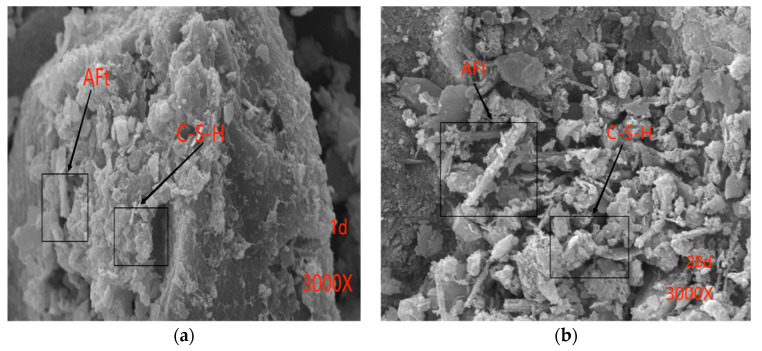
Scanning electron microscopy images. (**a**) Hydration reaction time of GSC with a 70% RA content for one day; (**b**) Hydration reaction time of GSC with a 70% RA content for 28 days.

The experimental parameters for the scanning electron microscopy (SEM, Zeiss, Oberkochen, Germany) analysis were as follows: Figure 7a and Figure 8a were conducted with an Extraction High Tension (EHT) of 20 kV, a Working Distance (WD) of 6.5 mm, and a magnification of 3.00 k×. For Figure 7b and Figure 8b, the parameters were set to EHT = 20 kV, WD = 6.0 mm, and a magnification of 3.00 k×.

The microstructural characteristics of OPC and GSC were examined at various curing durations using scanning electron microscopy (SEM). Figure 7a,b presents SEM images depicting the microstructure of ordinary Portland cement after curing for 1 day and 28 days. In these images, the conspicuous white fibrous entities correspond to the C-S-H gel, while the needle-like formations represent the hydration product AFt, some of which are encased within the fibrous C-S-H gel. With the progression of hydration, AFt assumes a more robust morphology, accompanied by an augmented presence of the surrounding C-S-H gel, forming a closely intertwined matrix. Although discernible AFt is evident after the initial day of hydration, its morphology becomes more pronounced with the elapse of time.

Figure 8a,b provides SEM images illustrating the microstructure of GSC after curing for 1 day and 28 days. The hydration products of GSC closely resemble those of OPC, comprising a C-S-H gel and AFt. However, GSC exhibits a higher abundance of AFt, particularly at the 28-day hydration mark when ettringite (AFt) emerges as a principal contributor to early strength, notably in the context of sulfate-resistant cements. Furthermore, it is evident from Figure 7b and Figure 8b that after a curing period of 28 days, the hydration products of GSC are notably more abundant compared to those of OPC. Consequently, it can be inferred that the microstructure of GSC exhibits a higher degree of compactness in comparison to OPC. This microscopic analysis elucidates why the strength of GSC surpasses that of OPC.

## 4. Conclusions

In the pursuit of investigating the viability of substituting GSC for OPC in stabilizing RA base layers, and to elucidate the ensuing disparities in mechanical properties, the present study conducted a series of comprehensive experiments. These experiments encompassed compaction tests, unconfined compressive strength assessments, and indirect tensile strength analyses. Microscopic examinations, employing XRD and SEM, were also carried out. The principal findings are delineated as follows:

(1) Upon the substitution of GSC for OPC, a discernible augmentation in both the optimal moisture content and the maximum dry density, by approximately 0.3% and 0.1 g/cm^3^, respectively, was observed. Concurrently, the mechanical strength of the composite material exhibited enhancement. Specifically, the 7-day unconfined compressive strength witnessed an approximate 30% escalation, whereas the 28-day counterpart displayed an increase of about 25%. Moreover, the 7-day indirect tensile strength manifested an approximate 25% rise, with a corresponding increase of about 25% observed at the 28-day mark. The phenomenon observed by Zhu Lin [29] and Sun Zhengning [19] in their experiments is similar: the strength of gypsum slag cement is greater than that of ordinary Portland cement.
(2) At a recycled material content of 0% and a 4:1 slag–activator ratio, negligible strength was discerned, whereas at a ratio of 4:2, normal strength characteristics were observed. Notably, when the slag-to-activator ratio was 4:1, and the recycled material content was 50%, 60%, and 70%, consistent and heightened strength properties were manifested. XRD investigations and a thorough literature review unveiled that the alkalinity of the entire system is pivotal for the hydration reaction of gypsum slag cement. A salient distinction between recycled aggregate and natural aggregate lies in the alkaline substances present in recycled micro-powder, such as CaOH, which actively promote the hydration reaction of gypsum slag cement. Consequently, the simultaneous use of gypsum slag cement and recycled aggregate not only proves to be economically and environmentally judicious but also imparts an augmented mechanical strength to the semi-rigid base layer.
(3) XRD outcomes demonstrated that the hydration products of gypsum slag cement and ordinary Portland cement exhibited no substantive distinctions, both primarily consisting of ettringite and C-S-H gel. Furthermore, with the progression of curing time, the characteristic peaks of the ettringite and the C-S-H gel intensified. Intriguingly, the CaSO_4_ content in gypsum slag cement progressively diminished during the hydration reaction.
(4) SEM analysis has revealed that the hydration products obtained from cement and gypsum slag cement exhibit similarities. Nevertheless, under identical curing conditions, a discernible disparity arises in the production of ettringite. GSC manifests a greater quantity of ettringite compared to cement, accompanied by a denser microstructure. This observation elucidates the macroscopic phenomenon wherein the compressive strength and splitting tensile strength of gypsum slag cement surpass those of ordinary Portland cement.
(5) In conclusion, this study primarily investigates the mechanical strength of GSC, comparing it with OPC. Long-term and road performance aspects were not addressed in this paper. However, it should be noted that due to the higher optimal water content of GSC compared to OPC under similar conditions, the former requires a greater water content. Consequently, during drying shrinkage tests, GSC exhibits greater shrinkage compared to OPC. Moreover, a review of the literature suggests that GSC demonstrates poor frost resistance [30]. These findings indicate potential directions for future research on GSC.

## Figures and Tables

**Table 1:** OPC testing findings.

Indices		Dimensions	Technical Specifications	Actual Measurements
Specific surface area		m^2^/kg	≥300	339
Stability		mm	≤5.0	1.8
Initial Setting Time		min	≥45	294
Final Setting Time		min	≤600	390
LOI		%	≤5.0	4.18
Sulfur Trioxide		%	≤3.5	2.31
Magnesium Oxide		%	≤5.0	2.36
Chloride ion		%	≤0.06	0.041
3-day strength	Flexural strength	MPa	≥3.5	4.6
	Compressive strength	MPa	≥17.0	24.8
28-day strength	Flexural strength	MPa	≥6.5	8.9
	Compressive strength	MPa	≥42.5	50.7

**Table 2:** Chemical composition of OPC.

Composition	SiO_2_	Al_2_O_3_	Fe_2_O_3_	CaO	MgO	SO_3_	K_2_O	Na_2_O	LOI	f-CaO
Content (%)	21.10	5.23	3.37	61.72	2.25	1.88	0.72	0.40	2.55	0.36

**Table 3:** Chemical composition of blast furnace slag.

Composition	SiO_2_	Al_2_O_3_	CaO	MgO	SO_3_	Other
Content (%)	34.9	14.9	40.8	5.1	2.2	2.1

**Table 4:** Aggregate sieving test results.

Aggregate Type	Mass % Passing through the Following Sieves (Square Mesh, mm)												
	31.5	26.5	19.0	16.0	13.2	9.5	4.75	2.36	1.18	0.6	0.3	0.15	0.075
Recycled coarse aggregates	100	88.9	7.5	1.7	0.5	0	0	0	0	0	0	0	0
Recycled medium aggregates	100	100	100	94.7	81.1	63.2	2.2	0	0	0	0	0	0
Recycled fine aggregate	100	100	100	100	100	100	90.7	54.8	39.8	27.7	18.9	13.6	6.5
Natural coarse aggregate	100	100	36.7	16.7	4.5	0.7	0.6	0.2	0	0	0	0	0
Natural medium aggregate	100	100	100	100	97.5	56.9	4.3	2.2	2.1	2	0	0	0
Natural fine aggregate	100	100	100	100	100	100	100	83.5	63	52.1	42.1	27.8	20.5

**Table 5:** Performance test results of the NAs and RAs.

Indices	Limestone and Recycled Aggregates						
	0–5 mm Limestone	5–15 mm Limestone	15–25 mm Limestone	0–5 mm Recycled Aggregate	5–15 mm Recycled Aggregate	15–31.5 mm Recycled Aggregate	Regulatory Requirement
Needle-like content (%)	/	11.7	12.7	/	16.5	17.6	≤22
Crushing value (%)	/	18.7	19.4	/	24.3	25.6	≤26
Apparent density (g/cm^3^)	2.886	2.784	2.723	2.673	2.583	2.532	≥2.5
Water absorption (%)	2.3	0.91	0.85	12.3	6.69	5.27	

**Table 6:** The dense-graded gradation of the cement-stabilized materials.

Recycled Material Content	Percentage of Quality Adopted (%)						
	31.5	19.0	9.50	4.75	2.36	0.6	0.075
Grass-roots unit	100	68–86	38–58	22–32	16–28	8–15	0–3
0% Recycled Material Content	100	77.2	50.5	27.8	20.4	13.4	1.3
50% Recycled Material Content	100	76.6	54.1	27.3	17.9	10.5	1.6
60% Recycled Material Content	100	70.5	51.6	27.4	17.3	9.7	1.7
70% Recycled Material Content	100	68.8	48.5	27.3	17.3	9.7	1.7

**Table 7:** The compaction test parameters.

Weight of the Hammer (kg)	Diameter of the Hammer Face (cm)	Drop Height (cm)	Dimensions of the Test Cylinder			Number of Hammer Blows	Number of Hammer Blows per Layer	Average Unit Compaction Energy (J)	Permissible Maximum Nominal Particle Size (mm)
			Inner Diameter (cm)	Height (cm)	Volume (cm^3^)				
4.5	5.0	45	15.2	12.0	2177	3	98	2.677	37.5

**Table 8:** Optimum moisture content test results.

Binder Material Content/%	Optimal Moisture Content under Different RA Contents/%			
	0%	50%	60%	70%
3.5 (OPC)	4.6	6.8	7.1	7.8
4.5 (OPC)	4.9	7.2	7.4	8.1
5.5 (OPC)	5.2	7.4	7.6	8.3
3.5 (GSC)	5.1	7.2	7.5	8.2
4.5 (GSC)	5.3	7.4	7.7	8.4
5.5 (GSC)	5.5	7.6	7.9	8.6

**Table 9:** Maximum dry density test results.

Binder Material Content/%	Maximum Dry Density under Different RA Contents/(g/cm^3^)			
	0%	50%	60%	70%
3.5 (OPC)	2.394	2.211	2.179	2.104
4.5 (OPC)	2.416	2.213	2.198	2.154
5.5 (OPC)	2.451	2.243	2.212	2.184
3.5 (GSC)	2.501	2.297	2.267	2.214
4.5 (GSC)	2.523	2.332	2.297	2.255
5.5 (GSC)	2.549	2.376	2.325	2.296

**Table 10:** The compositions and weights of the specimens across different groups.

Binder Material Content/%	Weight of Various Components under Different RA Dosages											
	0%			50%			60%			70%		
	A/g	W/g	C/g	A/g	W/g	C/g	A/g	W/g	C/g	A/g	W/g	C/g
3.5 (OPC)	6006	286	210	5546	390	194	5466	402	191	5278	426	185
4.5 (OPC)	6003	307	270	5498	414	247	5461	422	246	5352	453	541
5.5 (OPC)	6032	331	332	5520	431	304	5444	436	299	5375	471	296
3.5 (GSC)	6274	331	220	5762	429	202	5687	441	199	5554	471	194
4.5 (GSC)	6269	347	282	5794	448	261	5707	459	257	5603	492	252
5.5 (GSC)	6273	364	345	5847	469	322	5722	477	315	5651	513	311

**Table 11:** Seven-day unconfined compressive strength test.

Binder Material Content/%	Unconfined Compressive Strength after Seven Days under Different RA Contents/MPa			
	0%	50%	60%	70%
3.5 (OPC)	3.9	3.7	3.5	3.2
4.5 (OPC)	5.3	4.9	4.7	4.5
5.5 (OPC)	6.2	5.8	5.4	5.1
3.5 (GSC)	4.8	4.6	4.3	4
4.5 (GSC)	6.6	6.1	5.8	5.6
5.5 (GSC)	7.7	7.2	6.7	6.3

**Table 12:** The 28-Day unconfined compressive strength test.

Binder Material Content/%	Unconfined Compressive Strength after 28 Days under Different RA Contents/MPa			
	0%	50%	60%	70%
3.5 (OPC)	4.3	4.1	3.9	3.5
4.5 (OPC)	5.8	5.4	5.2	4.9
5.5 (OPC)	6.8	6.4	5.9	5.6
3.5 (GSC)	5.3	5.1	4.7	4.4
4.5 (GSC)	7.2	6.7	6.4	6.1
5.5 (GSC)	8.5	7.9	7.4	6.9

**Table 13:** Indirect tensile strength after seven days.

Binder Material Content/%	Indirect Tensile Strength after Seven Days under Different RA Contents/MPa			
	0%	50%	60%	70%
3.5 (OPC)	0.32	0.29	0.25	0.21
4.5 (OPC)	0.38	0.34	0.32	0.29
5.5 (OPC)	0.42	0.39	0.35	0.33
3.5 (GSC)	0.38	0.34	0.30	0.25
4.5 (GSC)	0.46	0.41	0.38	0.35
5.5 (GSC)	0.51	0.46	0.42	0.36

**Table 14:** Indirect tensile strength after 28 days.

Binder Material Content/%	Indirect Tensile Strength after 28 Days under Different RA Contents/MPa			
	0%	50%	60%	70%
3.5 (OPC)	0.35	0.32	0.28	0.23
4.5 (OPC)	0.42	0.37	0.35	0.32
5.5 (OPC)	0.46	0.43	0.39	0.33
3.5 (GSC)	0.42	0.37	0.33	0.28
4.5 (GSC)	0.51	0.45	0.42	0.38
5.5 (GSC)	0.56	0.51	0.46	0.39

## Data Availability

Data are contained within the article.
